# Severe Respiratory Infections in Rheumatoid Arthritis Patients: An Observational Study of 528 Patients from a Single University Hospital

**DOI:** 10.3390/jcm14041174

**Published:** 2025-02-11

**Authors:** Lucía C. Domínguez-Casas, Iván Ferraz-Amaro, Santos Castañeda, Ricardo Blanco

**Affiliations:** 1 Rheumatology, Hospital Universitario Marqués de Valdecilla, Immunopathology Group-IDIVAL, 39008 Santander, Spain; 2 Rheumatology, Hospital Universitario de Canarias, 38320 Tenerife, Spain; iferrazamaro@hotmail.com; 3 Rheumatology, Hospital Universitario de La Princesa, IIS-Princesa, 28006 Madrid, Spain; scastas@gmail.com

**Keywords:** rheumatoid arthritis, infections, SRI, vaccination

## Abstract

Patients with rheumatoid arthritis (RA) have an increased risk of infections. This may be linked to disease-related factors, immunosuppressive therapy and the presence of comorbidities. **Background/Objectives:** In an unselected group of RA patients, our aims were to assess the following: (a) the incidence and (b) features of diseases and (c) the predictive factors of severe respiratory infection (SRI). **Methods:** An observational and retrospective study of all patients with RA included in the vaccination program of our hospital between October 2011 and October 2018 was conducted. The follow-up continued until December 2020. Patients with SRI, defined as those that required hospitalization or at least one dose of intravenous antibiotic treatment in the emergency room, were (a) compared with those not requiring hospital admission and (b) studied for predictive factors of SRI (multivariate analysis adjusted for age and sex). The vaccination program in our hospital includes vaccination against influenza, *S. pneumoniae* and *H. influenzae*. Information on the patients, infections and hospitalizations was retrospectively retrieved from the hospital and general physician records. **Results:** We studied 528 RA patients (409 women/119 men) with a mean age of 58.9 ± 13.2 years. A total of 55 patients (10.4%) suffered 89 SRIs. The median [IQR] number of hospitalizations per patient was 1.5 [1–2]. Patients with an SRI were older, had had RA for longer and had more comorbidities (hypertension, hypercholesterolemia, diabetes and interstitial lung disease). These patients had more ACPA positivity, more extra-articular manifestations and high disease activity at the time of their vaccination. Treatment with glucocorticoids, methotrexate and leflunomide was seen in a higher number of patients. Predictive factors for SRI were age; time of evolution of RA; associated comorbidities, especially hypertension and diabetes; extra-articular manifestations, especially interstitial lung disease; and treatment with glucocorticoids, methotrexate and leflunomide. **Conclusions:** Despite being included in a vaccination program, about 10% of our patients required hospitalization due to an SRI. The main predictive factors were certain comorbidities, interstitial lung disease and treatment with glucocorticoids. Predicting SRI in RA patients remains an unmet need.

## 1. Introduction

Rheumatoid arthritis (RA) is a chronic inflammatory disease characterized by polyarthritis that produces progressive joint damage [1]. Furthermore, RA is associated with increased mortality, mainly due to high cardiovascular risk, malignancies and infections [2]. This augmented risk is due to the disturbance of the immune system’s function, co-morbidities and, especially, treatment with glucocorticoids (GCs) and disease-modifying anti-rheumatic drugs (DMARDs) [3,4,5]. However, old cohorts of patients with RA from the pre-biological-treatment era have demonstrated that the main markers of infection risk are the severity of the disease and the presence of comorbidities [1,3]. In 2002, Doran et al. performed a study on 609 patients with RA that compared them to 609 healthy individuals and demonstrated that the incidence of infections and the number of admissions for infections were significantly higher in the first group (9.57/100 person-years vs. 5.09/100 person-years) [3].

Along the same lines, in a prospective observational study using data from the British Society for Rheumatology Biologics Register (BSRBR) of RA patients (BSRBR-RA), the risk of serious infections (SIs) among 11,798 TNF inhibitor (TNFi)-treated patients and 3598 non-biologic DMARD-treated patients was compared, and the results suggested that TNFi therapy was associated with a small but significant overall risk of SI (20%) [6]. Registries for other biologic agents targeting alternative immune pathways have comparable safety data to anti-TNF drugs [7,8].

The most frequent SIs in RA are respiratory tract infections, with *Streptococcus pneumoniae* being the most common causative microorganism [9,10].

In this line of research, the BSRRBR-RA has revealed that SIs in RA patients are frequent (with an annual rate of a first SI of 14%), with serious respiratory infections (SRIs) being the most prevalent (44% of all events) and recurring each year, with the highest risk observed within the initial 12-month period after an infection. In fact, subjects who experienced sepsis had the highest risk (19.7%) of developing subsequent SIs in the following 12 months [10].

Considering all this, the aim of this study was to assess the incidence, features of disease and predictive factors of SRI in the real-world context of patients with RA and to evaluate the influence of a vaccination program on SRI incidence and severity.

## 2. Materials and Methods

### 2.1. Design and Enrollment Criteria

We performed a retrospective study of patients diagnosed with RA from a single center (Marqués de Valdecilla University Hospital) that were consequently included in the vaccination program of the hospital from over a seven-year period (from October 2011 to October 2018). These patients were later followed up with until December 2020. RA was diagnosed according to the EULAR/ACR 2010 criteria [11]. Patients diagnosed before 2010 were re-evaluated and their diagnosis was confirmed with these criteria. Patients diagnosed with RA were referred to the Preventive Medicine department of our hospital when a decision to initiate biological treatment was made. The vaccination program of our hospital during this period (2011–2018) included influenza, pneumococcal and *Haemophilusinfluenzae* vaccines. All patients in our study received these three vaccines. Other vaccines recommended for patients based on their serologies included the hepatitis B virus vaccine, the human papillomavirus (HPV) vaccine, the MMR vaccine (measles, mumps and rubella) and the meningococcal C vaccine. The SARS-CoV-2 vaccine was not included in this study because it was introduced more than 2 years after the end date of the study (January 2021).

### 2.2. Outcome Variables

The primary outcome was the number of SRIs, defined as those that required hospitalization or at least one dose of intravenous antibiotic drug in the emergency room [12]. The number of admissions due to these infections, laboratory and microbiology data, and antibiotic treatments were also registered.

At baseline (date of first vaccination), predefined variables potentially related to the development of an SRI were evaluated. Demographic data (age, sex and RA duration) and co-morbid conditions (active smoking, hypertension, hypercholesterolemia and diabetes) were assessed to evaluate these risk factors.

The following variables related to the severity of RA were included: the presence of erosions and extra-articular manifestations, rheumatoid nodules, interstitial lung disease and associated Sjögren syndrome. In all patients, interstitial lung disease was diagnosed based on high-resolution computed tomography (HRCT) and a pulmonologist’s expert evaluation. Sjögren syndrome was diagnosed based on 2012 SICCA and 2016 EULAR/ACR criteria, depending on the year of RA diagnosis.

Laboratory findings included positivity for the rheumatoid factor (RF) and anti-citrullinated peptide antibodies (ACPA), as well as RA activity, as assessed by C-reactive protein (CRP) and erythrocyte sedimentation rate (ESR). The treatment of RA with glucocorticoids, conventional synthetic DMARDs (csDMARDs), biological DMARDs (bDMARDs) and JAK inhibitors (JAKinh) was also registered.

### 2.3. Data Collection and Statistical Analysis

Patients’ data were collected from the clinical records of our hospital (Hospital Universitario Marqués de Vadecilla) and from the General Practitioners’ database. First, data were reviewed to confirm the diagnosis and then stored in a computerized data file. The basal demographic and clinical characteristics of patients with RA were described as means ± standard deviation (SD) or as percentages for categorical variables. For non-normally distributed continuous variables, data were expressed as a median and interquartile range (IQR). The crude incidence rate of every infection was presented as a number of events per person-year. Multivariable Cox proportional hazards and recurrent events regression analyses were performed to assess the relationship between demographic and RA-disease-related data and infections. This analysis was adjusted for principal confounding factors such as sex, age and the presence of comorbidities. The confounders’ critical value was set to *p* = 0.2 in the model. All the analyses used a 5% two-sided significance level. Statistical analysis was performed by Stata software, version 17/SE (Stata Corp, College Station, TX, USA), with a *p*-value < 0.05 considered as statistically significant.

## 3. Results

### 3.1. Baseline Clinical Features of RA Patients at Inclusion

A total of 528 patients (409 women/119 men) diagnosed with RA were selected and included in the vaccination program of our hospital during this 7-year period (October 2011–October 2018). Follow-ups were conducted until December 2020. The main characteristics of all patients are summarized in Table 1.

The mean age of the patients was 58.9 ± 13.2 years. Most of those patients were women (77%). Almost half of the patients were active smokers (43%) and presented with co-morbidities such as hypertension (41%), hypercholesterolemia (43%) and diabetes (15%). Positivity for RF and ACPAs was 55% and 54%, respectively. Two hundred and seven patients presented with an erosive disease (39%). Regarding extra-articular manifestations, 25 patients presented with rheumatoid nodules (5%), 36 patients had associated Sjögren’s syndrome (7%) and 34 patients had RA-associated interstitial lung disease (6%).

At the time of vaccination, approximately 61% of patients were taking glucocorticoids, with an average dose of prednisone equivalent of 5 mg/day (range: 5–10 mg/day). Moreover, most of the patients were under treatment with csDMARDs. Methotrexate was the main csDMARD (61%), followed by leflunomide (9%) and sulfasalazine (3%). Biological therapy was administered in 134 patients (25%): etanercept (n = 46), adalimumab (38), tocilizumab (19), rituximab (11), infliximab (8), golimumab (8), certolizumab (2) and abatacept (2) (Table 1).

### 3.2. Clinical Characteristics of Severe Infections

Patients were followed-up with for an average period of 5 years (range: 2 months—9 years). During this period, 55 of these patients (10.4%) required at least one admission (89 admissions in total) due to an SRI, with a median [IQR] of 1.5 [1–2] per patient. The estimated annual rate was 2.5/100 person-years. As expected, most of the infections (60%) were diagnosed during the most common periods; 20% were diagnosed in autumn (September to November in Spain) and 40% in winter (December to March in Spain).

Only 33 respiratory infections were microbiologically proven (Table 2), 4 of which corresponded to bilateral SARS-CoV-2 pneumonia. During the follow-up period, 12 patients passed away due to an SRI, representing 21.8% of patients in our cohort.

### 3.3. Predictive Factors Associated with Serious Respiratory Infections

A Cox regression analysis was performed to identify potential risk factors for the development of an SRI. Table 3 summarizes the main predictive factors for SRI development in our cohort of patients with RA.

The analysis showed that age was one of the main risk factors for SRIs [Hazard Ratio (HR) 1.07; 95%CI: 1.05–1.10; *p* < 0.001]. In addition, the presence of comorbidities such as hypertension (HR 2.97; 95%CI: 1.68–5.22; *p* < 0.001), dyslipidemia (HR 1.81; 95%CI: 1.06–3.09; *p* = 0.031) and diabetes (HR 2.41; 95%CI: 1.33–4.38; *p* = 0.004) was also associated with the development of SRIs.

The analysis of RA features revealed that positivity for ACPAs (HR 1.76; 95%CI: 1.01–3.08; *p* = 0.046) and the presence of extra-articular manifestations (HR 2.28; 95%CI: 1.29–9.74; *p* = 0.004) were associated with infection risk. Furthermore, the results showed that patients with interstitial lung disease were five times more likely to experience an SRI, a statistically significant difference (HR 5; 95%CI: 2.57–9.74; *p* < 0.001). The same results were observed when adjusting these factors for age, sex and cardiovascular risk factors (current smoking, hypertension, dyslipidemia and diabetes). Finally, patients with higher RA activity at the time of their vaccination, evaluated by ESR, had a greater risk of suffering from an SRI (HR 1.02; 95%CI: 1.01–1.03; *p* = 0.001).

With respect to RA treatment, one of the main risk factors for the development of infections was the use of glucocorticoids and the length of a patient’s exposure to them. Patients undergoing corticosteroid treatment exhibited an almost threefold increased risk of SRI, with consistent results upon adjusting the analysis for age and cardiovascular risk factors. Furthermore, this risk was higher in patients with a longer exposure to GCs (HR 1.024; 95%CI: 1.006–1.042; *p* = 0.01).

The analysis of csDMARD treatments revealed that leflunomide was associated with a higher risk of SRI development (HR 2.07; 95%CI: 1.01–4.24; *p* = 0.004), as well as a long exposure time to this treatment (HR 1.01; 95%CI: 1.003–1.03; *p* = 0.015). Regarding methotrexate, it was observed that patients with SRIs had a longer exposure to it (HR 1.007; 95%CI: 1.002–1.01; *p* = 0.004). However, sulfasalazine did not show an association with the development of severe infections. Similar results were observed when adjusting for age, sex and cardiovascular risk factors.

The Cox regression analysis of treatment with bDMARDs and JAK inhibitors at baseline did not exhibit any specific relationship with SRI development. However, 24 patients (43%) were under biological treatment when diagnosed with an SRI.

### 3.4. Factors Associated with Recurrent Infections

In this study, 55 patients presented with 89 hospital admissions due to SRIs, meaning that recurrent infections were frequent in this group. In Table 4, a Cox regression analysis is shown to explore potential risk factors associated with the development of recurrent SRIs. The data analysis indicates that patients who experience more than one SRI tend to be older (HR 1.07; 95%CI: 1.05–1.10; *p* < 0.001), have a longer disease duration (HR 1.03; 95%CI: 1.02–1.05; *p* < 0.001) and suffer from comorbidities, especially hypertension (HR 2.93; 95%CI: 1.49–5.73; *p* = 0.002) and diabetes (HR 2.31; 95%CI: 1.23–4.36; *p* = 0.009).

Significant differences were also observed between the presence of extra-articular manifestations, interstitial lung disease, and positive ACPAs.

Treatment with glucocorticoids emerged as the primary risk factor among all the therapies for recurrent infections (HR 2.5; 95%CI: 1.28–4.88; *p* = 0.007), as did a long period of time under treatment with leflunomide (HR 1.02; 95%CI: 1.01–1.02; *p* < 0.001) and methotrexate (HR 2.01; 95%CI: 1.003–1.01; *p* = 0.017).

Similar results were found after adjusting these factors for age and cardiovascular risk factors (smoking, hypertension, dyslipidemia and diabetes).

## 4. Discussion

Rheumatoid arthritis is a chronic autoimmune disease characterized by systemic inflammation and joint destruction. For years, patients with RA have been known to have an increased susceptibility to infections compared to the general population, as well as a higher mortality [13,14,15].

During this 9-year follow-up study of an RA cohort in real-world conditions, 55 patients required 89 hospital admissions due to an SRI, with an incidence rate of 2.5/100 person-years. Indeed, this incidence is lower than that of other published cohorts. Out of these 55 patients, 12 (nearly 22%) passed away due to the infection.

The vaccination program in our hospital (Hospital Universitario Marqués de Valdecilla) included vaccines against *S. pneumoniae*, *H. influenzae* and influenza. Microbiological isolation was obtained in 33 of the 89 SRI cases, 21 of which were caused by a pathogen not covered by the vaccination program.

The factors contributing to this increased susceptibility include the underlying inflammatory state of RA, which can impair immune function, as well as age, the presence of comorbidities and the use of DMARDs, which further suppress the immune system [3,4,5]. Increased infection risk has been described for patients with cardiovascular risk factors, especially diabetes [16,17,18]. Among the analyzed cohort, diabetes has been shown to have a significant relationship with SRI and recurrent events, as well as other cardiovascular risks such as hypertension and dyslipidemia. Smoking has also been identified as a factor related to infectious diseases [19]. However, the results of this study did not find any relationship between patients’ infection risk and tobacco.

Our region is characterized by an elderly population, which may influence the development of different infections. Moreover, the presence of comorbidities is more likely in these aged patients.

There are no consistent results in the literature regarding the relationship between disease activity and infectious disease, but this is possibly due to the higher dosages of immunosuppressive drugs administered to patients presenting more active RA [20]. In fact, the results suggest that the elevated disease activity evaluated by ESR was associated with an increased risk of developing an SRI. In this way, patients with more aggressive RA are expected to have a higher incidence of infections and greater disease severity [18,21] due to greater immune system disruption and the early and more extensive use of glucocorticoids and immunosuppressant agents. Interstitial lung disease is one of the extra-articular manifestations most linked to the development of SRIs [22]. In the presented study, patients testing positive for ACPAs and exhibiting extra-articular manifestations tended to experience more hospitalizations due to SRIs. Notably, interstitial lung disease was found to significantly increase the risk of such infections, multiplying them by five, as well as increase the presence of recurrent events.

Treatment with glucocorticoids has been one of the main risk factors for infections among the several rheumatic diseases included RA [22,23,24,25,26,27,28]. In our cohort of RA patients, treatment with glucocorticoids tripled the risk of experiencing an SRI, even after adjusting the results for age and cardiovascular risk factors. Moreover, the risk was higher the longer the use of corticosteroids lasted.

Conventional synthetic DMARDs, especially methotrexate and leflunomide, represent the first-line therapy for RA. However their use leads to an increased risk of developing infections and increased hospital admissions [29]. Regarding MTX, its prolonged use was specially related to an increased risk of SRI in our cohort. In addition, treatment with leflunomide led to a significant increase in this risk, as did its prolonged use. In the study of recurrent infections, it can be observed that the data from both drugs were similar, which shows a relationship between the use of these drugs and the recurrence of these infections.

Despite multiple studies having shown that biological treatment and JAK inhibitors increase the risk of infection, in our study, surprisingly, baseline treatment with biological drugs did not show a relationship to SRIs and their recurrence [30,31,32,33]. However, 43% of patients in our cohort were under treatment with biologics when diagnosed with an SRI. The low incidence of SRI in our cohort could be explained by the exhaustive control had over patients undergoing biological treatment, since they were treated with antibiotics upon seeing the first symptoms of infection. 

Furthermore, the majority of biologics used in our cohort were TNFα inhibitors, mainly etanercept, and it is well known that anti-TNF agents suppress the response to vaccines less than other immunomodulatory agents. In fact, the use of TNF-blocking agents has been associated with a more favorable response to pneumococcal vaccination in patients with autoimmune diseases compared to other immunosuppressive medication such as methotrexate, azathioprine or rituximab [34].

Another interesting discussion point is related to the interruption of csDMARDs/bDMARDs in RA patients who are going to be vaccinated against influenza, *S. pneumoniae* or COVID due to the risk of an SRI [35,36,37,38]. Recently, several works and recommendations have emerged suggesting the convenience of carrying out a temporary withdrawal from some DMARDs, particularly methotrexate and rituximab, especially when RA is inactive and well controlled, that weigh up the risks of a disease flare when RA is unstable [38,39,40].

One of the main strengths of our study is that it is one of the first studies analyzing this type of infection and their potential reduction in incidence through a vaccination program in our country. This will allow for better organization of these protocols, considering that it is not only targeted treatments that put patients at risk of infection, but also glucocorticoid treatments (when they are maintained for a long time) and patient comorbidities, which are predisposing factors for infection. However, our cohort is relatively small and the follow-up period was not very long, so not all the main factors could be properly analyzed. Indeed, our vaccination program is specifically administered to patients before they start biological treatment, meaning they have more aggressive RA and have undergone a greater number of immunosuppressive therapies. In addition, the absence of data such as DAS 28 (Disease Activity Score), CDAI (Clinical Disease Activity Index) and HAQ (Health Assessment Questionnaire) responses in many of the electronic records has hindered us in determining a possible association between these parameters and the risk of infection. 

## 5. Conclusions

In conclusion, patients with RA are at increased risk of infections. Furthermore, severe infections requiring hospital admissions are more common in these patients, thus increasing mortality and altering the course of the disease due to the need to suspend immunosuppressive treatments when these patients are hospitalized. Vaccination plays a crucial role in infection prevention in this vulnerable population. In our vaccinated cohort, despite the fact that a non-small percentage of patients suffered from SRIs, the annual rate was lower compared to that of other published cohorts. One outcome of this study is that we should consider vaccination at the time of diagnosis rather than waiting for the initiation of biological therapy. This is being followed as current practice in our hospital. Our study shows that, in addition to immunosuppressive therapies, the following risk factors are associated with the development of infections: age, comorbidities, interstitial lung disease and seropositivity. Nevertheless, further research in this area remains necessary, including prospective and multicenter studies. These studies may include other comorbidities such as obesity, the presence of pulmonary pathologies other than RA-related interstitial lung disease and RA activity measures with specific activity scores. It may also be beneficial to focus research on the management of immunosuppressive treatments specifically when vaccines are administered. As, for example, in one recent study that observed a higher effectiveness of the COVID-19 vaccine in RA and psoriatic arthritis patients who had temporarily suspended MTX for 1–2 weeks [38]. 

The final goal of these studies would be to establish universal vaccination recommendations that could be applied to all patients with RA and other similar conditions.

## Figures and Tables

**Table 1:** Demographic and disease-related data in our cohort of patients with rheumatoid arthritis at the time of their vaccination (time of inclusion in the study).

Baseline Demographic and Disease-Related Data	RA Patients (n = 528)
Age (years) (mean ± SD)	58.9 ± 13.2
Time since evolution of RA (months) (mean ± SD)	8.1 ± 9.3
Male, n (%)	119 (22.5)
Active smokers, n (%)	227 (42.8)
Hypertension, n (%)	217 (40.9)
Diabetes mellitus, n (%)	80 (15.1)
Dyslipidemia, n (%)	225 (42.5)
Positive RF, n (%)	293 (55.3)
Positive ACPAs, n (%)	283 (53.4)
Erosions, n (%)	207 (39.1)
Associated Sjögren’s syndrome, n (%)	36 (6.8)
Interstitial lung disease, n (%)	34 (6.4)
Subcutaneous nodules, n (%)	25 (4.7)
Vasculitis, n (%)	23 (4.3)
CRP (mg/dL)	1.17 ± 2.25
ESR (mm/h)	21.4 ± 21
**Baseline RA treatment**	
Prednisone, n (%)	321 (60.8)
Dose of prednisone, mg/day	5 (5–10)
*Conventional synthetic DMARDs*	
Methotrexate	322 (60.8)
Leflunomide	50 (9.4)
Sulfasalazine	14 (2.6)
*Biological DMARDs*, n (%)	
Etanercept	46 (8.7)
Adalimumab	38 (7.2)
Infliximab	8 (1.5)
Golimumab	8 (1.5)
Certolizumab	2 (0.4)
Tocilizumab	19 (3.6)
Rituximab	11 (2.1)
Abatacept	2 (0.4)

Abbreviations (in alphabetical order): ACPAs: anti-citrullinated peptide antibodies; CRP: C reactive protein; DL (dL): deciliter; DMARDs: disease-modifying anti-rheumatic drugs; ESR: erythrocyte sedimentation rate; H (h): hour; MG (mg): milligram; MM (mm): millimeter; N (n): number; RA: rheumatoid arthritis; SD: standard deviation.

**Table 2:** Causal microorganisms of severe respiratory infections.

Microorganisms	Number of Patients
*Streptococcus pneumoniae*	6
*Haemophilusinfluenzae*	4
*SARS-CoV-2*	4
*Pseudomonas aeruginosa*	3
*Serratialiquefaciens*	2
Influenza B	2
Influenza A	2
*Staphylococcus aureus*	1
*Stenotrophomonamaltophilia*	1
*Raoultellaplanticola*	1
*Mycobacterium tuberculosis*	1
*Moraxella catharralis*	1
Parainfluenza 4	1
Adenovirus	1
Bocavirus	1
Coronavirus OC43	1
HSV-1	1

Abbreviations (in alphabetical order): SARS-CoV-2: severe acute respiratory syndrome coronavirus 2; HSV-1: herpes simplex virus type I.

**Table 3:** Risk factors for severe respiratory infections.

	Univariate		Multivariate *	
	HR (95%CI)	*p* Value	HR (95%CI)	*p* Value
**Basal demographic and disease-related data**				
Age	**1.07 (1.05–1.10)**	**<0.001**	-	-
Male sex	1.07 (0.56–2.04)	0.83	-	-
*CV risk factors*				
Active smoking	1.33 (0.78–2.27)	0.29	-	-
Hypertension	**2.97 (1.68–5.22)**	**<0.001**	-	-
Dyslipidemia	**1.81 (1.06–3.09)**	**0.031**	-	-
Diabetes mellitus	**2.41 (1.33–4.38)**	**0.004**	-	-
*RA features*				
RA duration (years)	1.02 (1.003–1.04)	0.02		
Positive RF	1.40 (0.81–2.44)	0.23		
Positive ACPAs	**1.76 (1.01–3.08)**	**0.046**	**1.96 (1.12–3.47)**	**0.019**
Erosions	1.7 (0.92–2.67)	0.099	1.57 (0.92–2.69)	0.097
Extra-articular manifestations	**2.28 (1.29–4.01)**	**0.004**	**2.10 (1.18–3.75)**	**0.012**
Interstitial lung disease	**5 (2.57–9.74)**	**<0.001**	**3.80 (1.84–7.83)**	**<0.001**
Sjögren’s syndrome	1.63 (0.65–4.09)	0.29		
Subcutaneous nodules	1.04 (0.32–3.33)	0.95		
Other extra-articular manifestations	1.62 (0.58–4.49)	0.35		
CRP at time of vaccination	1.05 (0.97–1.14)	0.20	1.01 (0.93–1.09)	0.82
ESR at time of vaccination	**1.02 (1.01–1.03)**	**0.001**	1.81 (0.998–1.02)	0.11
**RA treatment at the time of inclusion**				
Prednisone	**2.62 (1.38–4.98)**	**0.003**	**2.54 (1.33–4.85)**	**0.005**
Time on prednisone (months)	**1.024 (1.006–1.042)**	**0.01**	1.016 (0.997–1.036)	0.10
Dose of prednisone	0.98 (0.90–1.08)	0.77		
*Conventional synthetic DMARDs*				
Methotrexate	0.61 (0.36–1.05)	0.075	0.64 (0.37–1.09)	0.097
Time on methotrexate (months)	**1.007 (1.002–1.01)**	**0.004**	1.004 (0.99–1.008)	0.14
Dose of methotrexate	0.98 (0.93–1.05)	0.73	0.99 (0.95–1.03)	0.69
Leflunomide	**2.07 (1.01–4.24)**	**0.004**	**2.21 (1** **.** **03–4.36)**	**0.042**
Time on leflunomide (months)	**1.01 (1.003–1.03)**	**0.015**	**1.02 (1.002–1.039)**	**0.027**
Dose of leflunomide	0.95 (0.82–1.10)	0.53		
Sulfasalazine	1.49 (0.36–6.15)	0.57		
*Biological DMARDs*				
Any biological treatment	0.67 (0.35–1.27)	0.22		
Etanercept	1.02 (0.43–2.39)	0.97		
Adalimumab	0.58 (0.18–1.87)	0.36		
Infliximab	1.01 (0.14–7.29)	0.99		
Golimumab	-			
Certolizumab	-			
Tocilizumab	0.87 (0.21–3.57)	0.85		
Rituximab	0.74 (0.10–5.36)	0.77		
Abatacept	-			

Abbreviations: ACPAs: anti-citrullinated peptide antibodies; CRP: C-reactive protein; CV: cardiovascular; DMARDs: disease-modifying anti-rheumatic drugs; HR: hazard ratio; RA: rheumatoid arthritis; RF: rheumatoid factor. * Adjusted by age, sex and CV risk factors (hypertension, dyslipidemia and diabetes). Significant values (*p* < 0.05) are in bold.

**Table 4:** Cox regression analysis of recurrent infectious events.

	Univariate		Multivariate *	
	HR (95%CI)	*p* Value	HR (95%CI)	*p* Value
**Basal demographic and disease-related data**				
Age	**1.07 (1.05–1.10)**	**<0.001**		
Male sex	1.13 (0.60–2.15)	0.70		
*CV risk factors*				
Active smoking	1.70 (0.96–3.06)	0.71		
Hypertension	**2.93 (1.49–5.73)**	**0.002**		
Dyslipidemia	1.68 (0.93–3.07)	0.087		
Diabetes mellitus	**2.31 (1.23–4.36)**	**0.009**		
*RA features*				
RA duration (years)	**1.03 (1.02–1.05)**	**<0.001**	**1.03 (1.01–1.05)**	**0.005**
Positive RF	1.08 (0.77–1.51)	0.65		
Positive ACPAs	**1.77 (0.98–3.18)**	**0.05**	**1.93 (1.11–3.33)**	**0.019**
Erosions	1.65 (0.92–2.97)	0.09	1.62 (0.89–2.94)	0.12
Extra-articular manifestations	**2.31 (1.91–4.46)**	**0.01**	**2.28 (1.26–4.15)**	**0.007**
Interstitial lung disease	**5.64 (2.54–12.55)**	**<0.001**	**4.63 (1.99–10.74)**	**<0.001**
Sjögren’s syndrome	1.29 (0.52–3.23)	0.58		
Subcutaneous nodules	0.88 (0.27–2.79)	0.83		
Other extra-articular manifestations	0.95 (0.36–2.49)	0.93		
CRP at time of vaccination	1.04 (0.96–1.11)	0.34		
ESR at time of vaccination	**1.02 (1.008–1.03)**	**<0.001**	1.01 (0.99–1.02)	0.09
**RA treatment at the time of inclusion**				
Prednisone	**2.5 (1.28–4.88)**	**0.007**	**2.41 (1.24–4.71)**	**0.01**
Time on prednisone (months)	**1.003 (0.99–1.006)**	**0.05**	1.00 (0.99–1.004)	0.77
Dose of prednisone	1.005 (0.93–1.08)	0.90		
*Conventional synthetic DMARDs*				
Methotrexate	0.58 (0.32–1.05)	0.07	0.59 (0.32–1.07)	0.083
Time on methotrexate (months)	**1.01 (1.003–1.01)**	**0.001**	**1.005 (1.0007–1.01)**	**0.025**
Dose of methotrexate	0.98 (0.91–1.05)	0.53		
Leflunomide	1.45 (0.72–2.92)	0.29		
Time on leflunomide (months)	**1.02 (1.01–1.02)**	**<0.001**	**1.02 (1.01–1.03)**	**0.006**
Dose of leflunomide	0.99 (0.74–1.32)	0.95		
Sulfasalazine	1.15 (0.33–4.01)	0.82		
*Biological DMARDs*				
Any biological treatment	0.54 (0.28–1.06)	0.07	0.61 (0.32–1.18)	0.14
Etanercept	0.84 (0.37–1.88)	0.66		
Adalimumab	0.60 (0.18–1.93)	0.39		
Infliximab	0.66 (0.10–4.31)	0.67		
Golimumab	-			
Certolizumab	-			
Tocilizumab	0.63 (0.15–2.55)	0.52		
Rituximab	-	-		
Abatacept	-	-		

Abbreviations: ACPAs: anti-citrullinated peptide antibodies; CRP: C-reactive protein; CV: cardiovascular; DMARDs: disease-modifying anti-rheumatic drugs; HR: hazard ratio; RA: rheumatoid arthritis; RF: rheumatoid factor. * Adjusted by age, sex and CV risk factors (hypertension, dyslipidemia and diabetes). Significant values (*p* < 0.05) are in bold.

## Data Availability

Data are unavailable due to privacy restrictions.
